# Adsorption Kinetics at Silica Gel/Ionic Liquid Solution Interface

**DOI:** 10.3390/molecules201219833

**Published:** 2015-12-10

**Authors:** Jolanta Flieger, Małgorzata Tatarczak-Michalewska, Anna Groszek, Eliza Blicharska, Ryszard Kocjan

**Affiliations:** Department of Analytical Chemistry, Faculty of Pharmacy with Division of Medical Analytics, Medical University of Lublin, 4a Chodźki St., Lublin PL-20093, Poland; m.tatarczak@op.pl (M.T.-M.); a.groszek@onet.eu (A.G.); bayrena@tlen.pl (E.B.); ryszard.kocjan@umlub.pl (R.K.)

**Keywords:** ionic liquids, silica gel, sorption kinetics, pseudo-second-order equation

## Abstract

A series of imidazolium and pyridinium ionic liquids with different anions (Cl^−^, Br^−^, BF_4_^−^, PF_6_^−^) has been evaluated for their adsorption activity on silica gel. Quantification of the ionic liquids has been performed by the use of RP-HPLC with organic-aqueous eluents containing an acidic buffer and a chaotropic salt. Pseudo-second order kinetic models were applied to the experimental data in order to investigate the kinetics of the adsorption process. The experimental data showed good fitting with this model, confirmed by considerably high correlation coefficients. The adsorption kinetic parameters were determined and analyzed. The relative error between the calculated and experimental amount of ionic liquid adsorbed at equilibrium was within 7%. The effect of various factors such as initial ionic liquid concentration, temperature, kind of solvent, kind of ionic liquid anion and cation on adsorption efficiency were all examined in a lab-scale study. Consequently, silica gel showed better adsorptive characteristics for imidazolium-based ionic liquids with chaotropic anions from aqueous solutions in comparison to pyridinium ionic liquids. The adsorption was found to decrease with the addition of organic solvents (methanol, acetonitrile) but it was not sensitive to the change of temperature in the range of 5–40 °C.

## 1. Introduction

Ionic liquids (ILs) are a broad class of salts melting at or below 100 °C. Over the last few years they have gained immense popularity in various fields of chemistry thanks to their environmentally friendly properties and the opportunities of matching their structure to a particular purpose. Initially, ionic liquids were used as reaction media for organic synthesis and biphasic catalysis primarily on industrial scale as an alternative to organic solvents [1,2,3,4,5]. So far different organic reactions like esterification, transesterification, nitration, and acetylation have been carried out using ionic liquids [6,7,8,9,10,11,12,13]. The high yields of all the above mentioned reactions indicate that ionic liquids possess huge potential in dedicated technologies of interest to the chemical industry. Currently increasing interest can also be observed in the use of ionic liquids on an analytical scale [14,15,16]. So far, ionic liquids have found a number of beneficial applications in electrochemistry [17,18,19,20,21,22,23,24,25] and separation techniques. There are examples of ionic liquid applications in the extraction of both ionic inorganic compounds, for instance metal cations [26], organic compounds [27] and biomolecules like peptides and proteins [28]. The leading role in the liquid-liquid extraction, even in a miniaturized version called liquid phase microextraction (LPME), is played by water-insoluble ionic liquids. In turn, the hydrophilic ionic liquids are used to create aqueous biphasic systems (ABS) in the presence of highly hydrated inorganic salts with kosmotropic (salting-out) properties. Such two phase systems are usually used for extractions, as an alternative to traditional liquid-liquid or liquid-solid partition systems. The resulting extraction system is especially suitable for the analysis of aqueous samples, and the use of the ABS technique for the extraction of hormones, alkaloids, vitamins, antibiotics from biological and environmental samples has been described [29,30,31,32,33,34,35].

The thermomorphic behavior of some ionic liquids allows carrying out the so-called homogenous liquid-liquid extraction (HLLE), wherein the phase separation is induced by temperature changes.

In recent years, attempts have been made to use ionic liquids to modify adsorbents by immobilization onto silica or polymeric supports [36,37,38,39]. The resulting so-called supported ionic liquid phases (SILPs) are used as sorptive materials in solid-phase extraction techniques. The first adsorbent subjected to modification was silica gel with immobilized 1-butyl-3-methylimidazolium hexafluorophosphate, which was further applied to the isolation of metals from aqueous media [40].

The aim of this research is the study of the adsorption process of imidazolium and pyridinium ionic liquids with different anions (Cl^−^, Br^−^, BF_4_^−^, PF_6_^−^) on silica gel. The influence of the kind of solvent, temperature, and the kind of anion and cation on adsorption efficiency were all examined. The adsorption mechanism of the examined ionic liquids with anions of different chaotropicity was studied with a pseudo-second-order kinetic model.

## 2. Results and Discussion

### 2.1. HPLC Conditions for Ionic Liquids Determination

There exist only a few papers dealing with high-performance liquid chromatography methods suitable for IL quantification [41,42,43,44]. Cations derived from ionic liquids can be analyzed separately in reversed-phase mode on different stationary phases. However, when using conventional octadecyl bonded phases with two component organic-aqueous mobile phases, the efficiency and separation selectivity tend to be poor.

**Table 1:** Structures of the investigated ionic liquids.

**BMIM PF_6_**	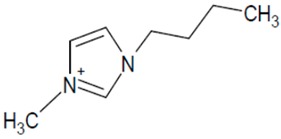	[PF_6_]^−^
**BMIM Cl**	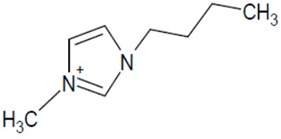	[Cl]^−^
**EMIM PF_6_**	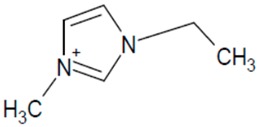	[PF_6_]^−^
**EMPyr Br**	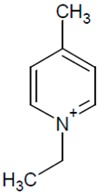	[Br]^−^
**EPyr BF_4_**	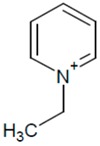	[BF_4_]^−^

It was proved that a significant improvement of peak shape and selectivity can be achieved by addition of acidic buffers and small amounts of a chaotropic salt to the mobile phase. The investigated ionic liquids (Table 1) have been analyzed on a Zorbax Extend-C18 (150 mm × 4.6 mm I.D., 5 μm) column using multicomponent mobile phases. The composition of eluent systems has been chosen according to the IL cation structure (polarity). The mobile phase components together with obtained peak parameters are collected in Table 2.

**Table 2:** The mobile phase components suitable for HPLC analysis of appropriate ionic liquids on a Zorbax Extend-C18 column.

Ionic Liquid	The Mobile Phase Composition	RT (min)	*k*	*A*s	N (EUP)	λ_max_
**BMIM PF_6_**	15%MeOH, 30 mM phosphate buffer, 30 mM NaBF_4_	3.87	1.98	1.73	38,480	220
**BMIM Cl**	15%MeOH, 30 mM phosphate buffer, 30 mM NaBF_4_	3.92	2.02	1.11	26,233	220
**EMIM PF_6_**	5%MeOH, 50 mM phosphate buffer, 30 mM NaPF_6_	3.20	1.46	1.32	12,673	220
**EMPyr Br**	8%MeOH, 30 mM phosphate buffer, 30 mM NaPF_6_	4.24	2.26	1.36	21,626	255
**EPyr BF_4_**	5%MeOH, 50 mM phosphate buffer, 30 mM NaPF_6_	2.61	1.01	1.34	20,300	255

The following equation was used to calculate the number of theoretical plates (N) according to USP standards: N = 16(RT/w)^2^, where RT is the actual full retention time of the appropriate peak, w is the peak width obtained by drawing tangents to each side of the peak and calculating the distance between the two points where the tangents meet the baseline. The tailing factor (*A*_s_) is based on the measurement of the half-width parameters A and B at 5% of the peak height, and is calculated as *A*_s_ = 1/2(1 + B/A). The detection was set at wavelength (λ_max_) according to the recorded spectra. The retention factor *k* is expressed as: (RT − t_0_)/t_0_ where t_0_ is the retention time of void volume marker.

**Figure 1:**
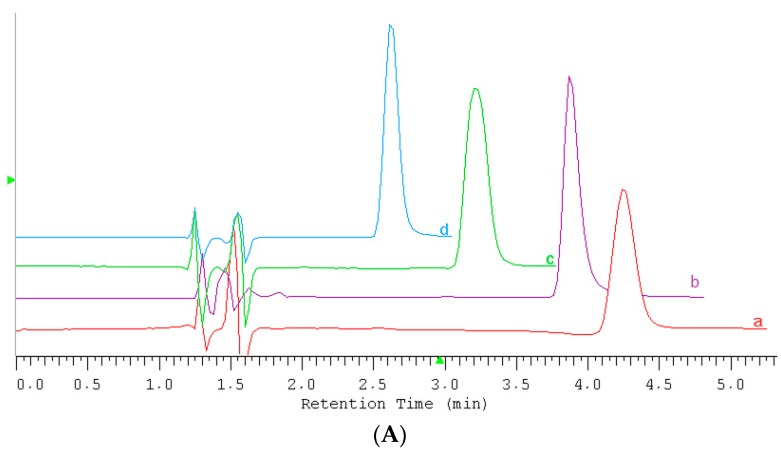

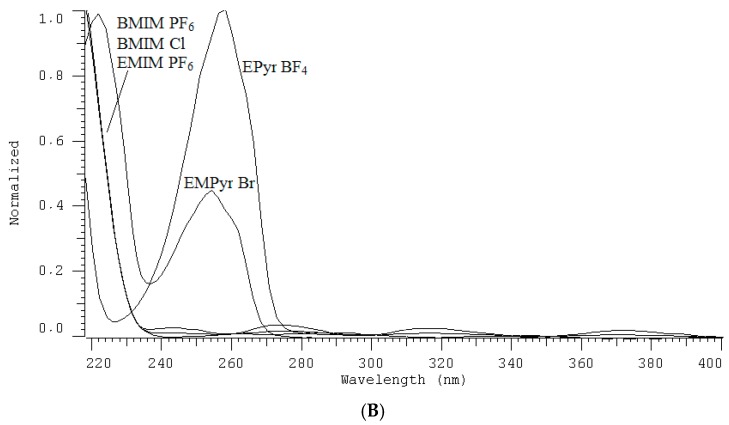
(**A**) Comparison of peaks: a—EMPyr Br, b—BMIM PF6, c—EMIM PF6, d—EPyr BF4 obtained on a Zorbax Extend-C18 column using the mobile phases listed in Table 2; (**B**) UV spectra obtained for the investigated ionic liquids: EMPyr Br, BMIM PF_6_, EMIM PF_6_, EPyr BF_4_.

As it can be seen, there is no significant difference in the retention times between ILs differing only in the kind anion (*cf.* BMIM Cl and BMIM PF_6_). The difference in retention times (3.92 − 3.87 = 0.04 min) is within the uncertainty in the measurements. Therefore, in subsequent figures (Figure 1A,B), only a kind of cation was illustrated.

The detection of the peaks was set at an appropriate wavelength chosen according to the recorded spectra in the range from 220 nm to 400 nm illustrated in Figure 1B.

### 2.2. Conditions for IL Quantification

The quantitative analysis of the examined ionic liquids was performed by the use of an external standard method applying the chromatographic system described in Section 2.1. A 20 µL sample of each dilution was injected in triplicate. The mean peak areas were taken for the construction of the calibration curves. The data were analyzed by a linear regression least squares model. The equation parameters for the regression lines are collected in Table 3.

**Table 3:** Linearity (*y* = a*x* + b), LOD, LOQ parameters for the investigated ionic liquids.

Ionic Liquid	Conc. Range: (µg·mL^−1^)	a ± SD	b ± SD	*R*^2^	s	F	LOD (µg·mL^−1^)	LOQ (µg·mL^−1^)	*n*
**BMIM PF_6_**	0.5–50	6824.04 (±98.07)	8521.04 (±2735.60)	0.9984	5397.32	4842.17	0.0474	0.1436	8
**BMIM Cl**	2.5–50	8110.84 (±145.70)	18027.91 (±3908.27)	0.9981	6575.45	3098.93	0.0593	0.1796	6
**EMIM PF_6_**	5–50	6482.15 (±108.17)	2386.59 (±3372.33)	0.9983	4833.93	3590.78	0.0551	0.1669	6
**EMPyr Br**	5–50	17059.98 (±333.56)	10599.09 (±10398.61)	0.9977	14905.46	2615.88	0.0645	0.1954	6
**EPyr BF_4_**	5–50	11531.70 (±126.90)	−4670.56 (±3956.14)	0.9993	5670.77	8257.59	0.0363	0.1100	6

Eight or six point calibration relationships were of excellent linearity, as expressed by the correlation coefficients (*R*^2^) higher than 0.9977 and high values of F—Fisher’s test. The limit of detection (LOD) and quantification (LOQ) were based on the calibration curves. The standard deviation of intercepts of regression lines was used as the standard deviation (SD). According to ICH requirements, LOD can be calculated as 3.3 SD of regression line/slope and LOQ as 10 SD of regression line/slope [45].

### 2.3. Influence of Ionic Liquid Kind and Concentration on Adsorption Efficiency

**Figure 2:**
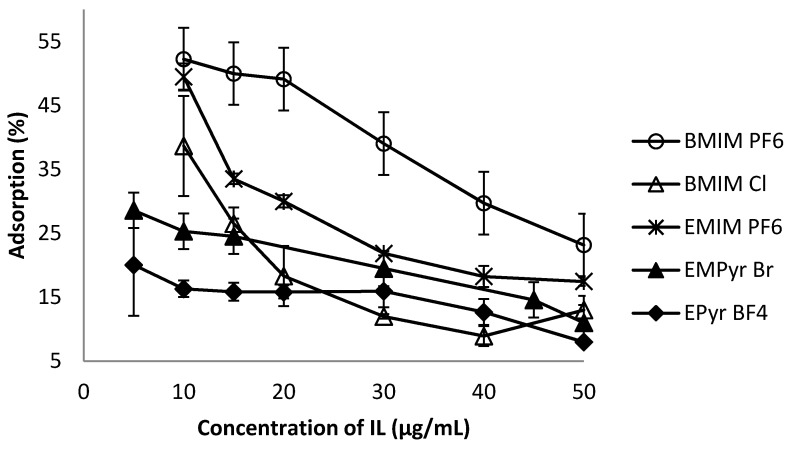
Influence of ionic liquid concentration on adsorption efficiency.

This study indicates that the absolute adsorption is higher for imidazolium ionic liquids in comparison to pyridinium ones (Figure 2). With increasing concentrations of ionic liquid in aqueous solutions from 10 to 50 µg·mL^−1^ for imidazolium and from 5 to 50 µg·mL^−1^ for pyridinium ionic liquids their adsorption efficiency decreases constantly almost half of the entire value. Coating the silica gel surface by ionic liquids ions is definitely enhanced by chaotropic anions. Summarizing, the order of ionic liquids regarding the percentage of their adsorption on silica gel increases from BMIM PF_6_ > EMIM PF_6_ > BMIM Cl to the remaining pyridinium cations: EMPyr > EPyr. In the case of pyridinium ionic liquids, the kind of anion is less significant in terms of adsorption capacity. Considering the fact that the ionic liquids at the beginning have the imidazolium cation but different anions, their adsorption ability would be affected mostly by the nature of anions.

Hexafluorophosphates (ΔG_hyd_ = −214 kJ/mol) are characterized by a more positive Gibbs free energy of hydration of the ions (ΔG_hyd_) in comparison to chlorides (ΔG_hyd_ = −347 kJ/mol) favoring electrostatic interactions in aqueous solution. Furthermore, ionic viscosity *B* coefficients of the Jones Dole equation (more positive for chlorides) differ significantly if comparing anions [46]. Thus the trend for the adsorption ability of these ionic liquids is in agreement with the order of the ΔG_hyd_ values and viscosity of the associated counterions.

### 2.4. Influence of Solvent Kind and Concentration on Adsorption Efficiency

Different solvents were investigated: pure water and water mixed with organic additives (methanol, acetonitrile). The adsorption efficiency was the highest for pure water and decreases constantly after addition of an organic solvent. Generally addition of 5% of organic solvent to water causes an adsorption efficiency decrease of about 5%, so pure water was adopted as solvent in further experiments.

**Figure 3:**
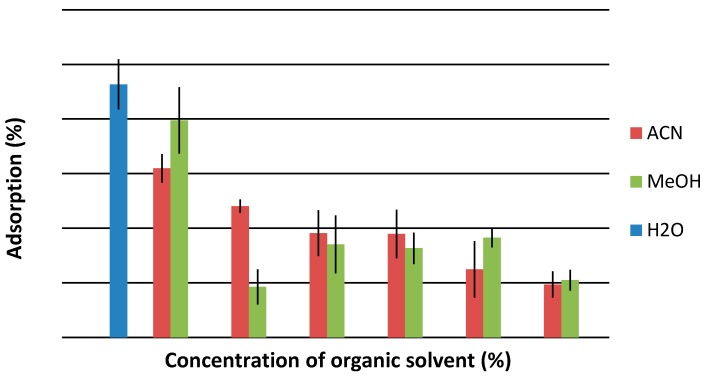
Influence of solvent kind and concentration on adsorption efficiency of 20 µg BMIM PF_6_ on 0.02 g of silica gel.

### 2.5. Influence of Temperature on Adsorption Efficiency

**Figure 4:**
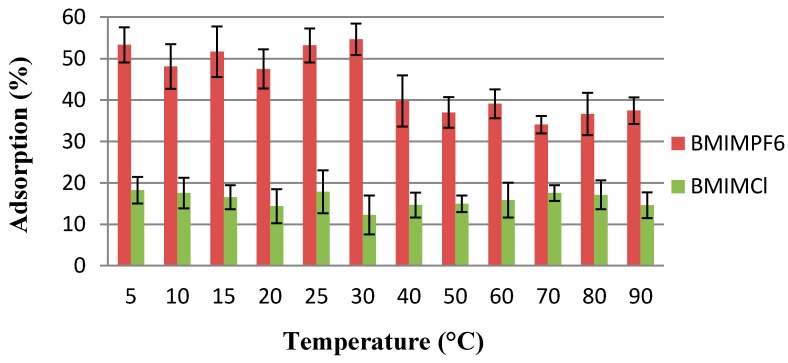
Influence of temperature on adsorption efficiency of BMIM PF_6_ and BMIM Cl.

It is common knowledge that temperature can be an important parameter influencing adsorption processes. Here, two imidazolium derivatives (BMIM PF_6_ and BMIM Cl) were used as representative ionic liquids to evaluate the effect of temperature on the adsorption effectiveness (Figure 4). The percentage of adsorption was determined in the range from 5 to 90 °C, and found to be in the range of measurement errors for both liquids up to 40 °C. At higher temperature, lowering of adsorption capacity was observed for ionic liquid with polyfluorinated anions indicating its possible decomposition. Simultaneously, this reflects a huge role of this anion in the adsorption process. The obtained results clearly indicate that in the temperature in the range of: 5–40 °C, the adsorption of ionic liquids is not sensitive to the temperature of the system. Therefore, the adsorption can be performed at room temperature, which is important in practice.

### 2.6. Kinetics of Adsorption Process

**Figure 5:**
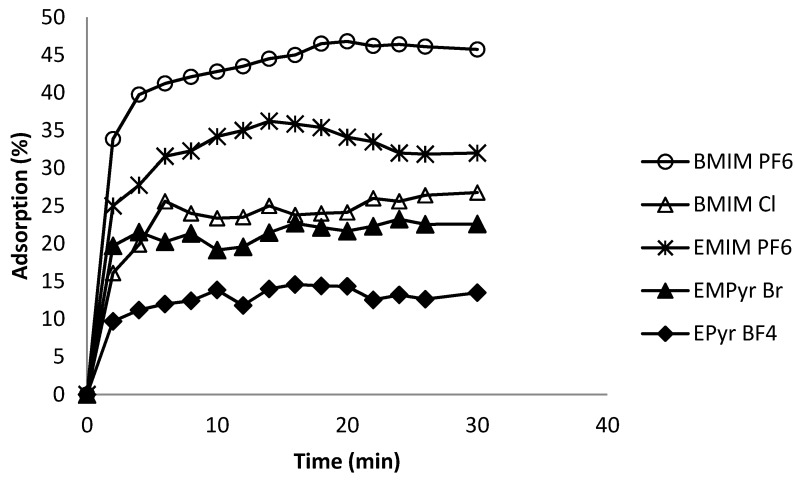
Effect of time on adsorption efficiency.

The phenomenon of adsorption at the solid/liquid interface plays a crucial role in processes applied on an industrial scale. The study of this phenomenon consists in analyzing the state of the adsorption equilibrium. Kinetic studies were conducted under optimum conditions determined in the preliminary experiments (initial concentration of ionic liquid 20 μg·mL^−1^, solution volume 2 mL, adsorbent mass 20 mg, temperature 25 °C). For the purpose of evaluating the effect of time on the adsorption efficiency, the time range from 0–30 min. was tested. Figure 5 shows that the adsorption efficiency gradually increased up to 5 min. In the region from 5 to 30 min. a type of saturation effect was observed, where no other significant changes in adsorption with time were observed.

Description of kinetic processes provides empirical or semi-empirical equations such as pseudo-first-order or pseudo-second-order. The pseudo-second-order equation which best fits the experimental data has been proposed by Ho *et al.* [47,48] and Blanchard [49]:

$$\frac{dq(t)}{dt}=k_{2}{\left(q_{e}-q(t)\right)}^{2}$$

**Figure 6:**
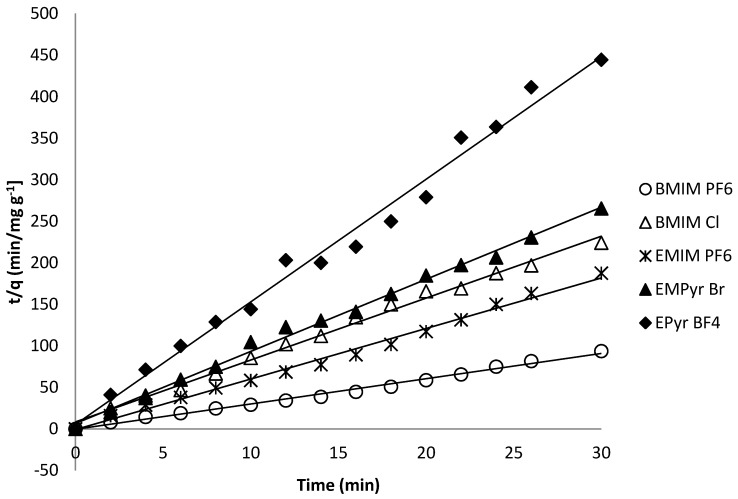
The linearized form of the pseudo-second-order kinetic equation.

Assuming *q*(*t* = 0) = 0, the linearized form of the above equation is the following one:

$$\frac{t}{q(t)}=\frac{1}{k_{2}{q_{e}}^{2}}+\frac{t}{q_{e}}$$

where *q_e_* is the amount of the solute (ionic liquid) adsorbed at equilibrium (mg/g), *k*_2_ (g·mg^−1^·min.) is the equilibrium rate constant of pseudo-second-order model. The uptake of the adsorbate at time *t*, *qt* (mg/g) was calculated by the following equation:

$$q_{t}=V\frac{c_{0}-c_{t}}{m}$$

where *c_t_* is the concentration of the ionic liquid in the solution at time *t*. The *q_e_* and *k_2_* values were determined from the slope and the intercept of the curves of *t/q vs. t*. Figure 6 shows the linearized form of the pseudo-second-order kinetic model. The determined kinetic parameters are shown in Table 4. As it can be seen, the correlation coefficients (*R*^2^), are considerably high, reinforcing the applicability of pseudo-second-order kinetic model. Furthermore, the calculated and experimental *q* values were very close to each other, giving Δ*q* (%) smaller than 7%. All these confirm the pseudo-second-order model of ionic liquids adsorption on silica gel indicating the strong physisorption as dominating the adsorption mechanism.

**Table 4:** Kinetic parameters for ionic liquids adsorption onto silica gel at 25 °C.

Ionic Liquid	Slope	Intercept	*R*^2^	*q*_e_	*k_2_*	∆*q* (%) ^1^	*e_r_* (%) ^2^	*q*_exp_
BMIM PF_6_	2.1272	1.1717	0.9989	0.470	3.862	5.1	7.3	0.232
BMIM Cl	7.4047	7.3303	0.9950	0.135	7.479	7.0	9.9	0.121
EMIM PF_6_	5.8594	1.1926	0.9905	0.170	28.788	6.2	9.3	0.160
EMPyr Br	8.7030	6.2926	0.9934	0.114	12.036	3.2	4.6	0.215
EPyr BF_4_	15.0048	5.3759	0.9855	0.066	41.880	6.1	8.7	0.069

^1^ $\Delta q(\%)=\sqrt{\frac{{\left[(q_{\text{exp}}-q_{cal})/q_{\text{exp}}\right]}^{2}}{N-1}}x100$; ^2^ $e_{r}(\%)=100\frac{|q_{\text{exp}}-q_{cal}|}{q_{\text{exp}}}$.

## 3. Materials and Methods

### 3.1. Reagents

Investigated compounds (Table 1) were obtained from Sigma (St. Louis, MO, USA) except for 1-ethyl-3-methylimidazolium hexafluorophosphate (EMIM PF6), which was from Fluka (Sigma-Aldrich Group, Lausanne, Switzerland). HPLC gradient-grade acetonitrile (ACN) and methanol (MeOH) were purchased from Merck (Darmstadt, Germany). Silica gel (LiChrospher Si 1000, mean particle size 10 µm) used as adsorbent was obtained from Merck. Prior to the adsorption process, the adsorbent was washed with distilled water to eliminate impurities, dried at 120 °C for 2 h. HPLC water was obtained from a Barnstead Deionising System (Dubuque, IA, USA). All mobile phases were buffered by the phosphate buffer (pH: 2.9–3.0). Its concentration was 30 or 50 mmol·L^−1^ in the whole mobile phase. The eluents were prepared by mixing the buffer solution, organic solvent and appropriate amounts of sodium hexafluorophosphate, sodium tetrafluoroborate.

### 3.2. Calibration Solutions

The stock solutions of ionic liquids at concentration of 1.0 mg·mL^−1^ and the calibration solutions were prepared gravimetrically and stored in darkness at 4 °C in glass vials. The calibration curves representing the dependence of the peak area on the concentration were used to perform quantitative analysis.

### 3.3. HPLC Quantification

Experiments were performed using a Merck Hitachi LaChrom HPLC (Merck) model equipped with a diode array detector, L-7350 column oven and L-7612 solvent degasser. The columns (250 mm × 4.6 mm I.D.) were packed with 5-μm Zorbax Eclipse XDB C18 (Agilent Technology, Waldbronn, Germany) pore size: 80 Å, surface area: 189 m^2^/g; with void volume determined by the injection of thiourea. Retention data were recorded at a flow-rate of 1 mL·min^−1^. The column was thermostated at 25 ± 0.1 °C. The detection was set at wavelength chosen accordingly with the recorded spectra. Typical injection volumes were 20 μL.

### 3.4. Adsorption Experiments

Batch adsorption experiments were carried out by an accurately weighed amount of adsorbent (0.02 g). Known weight of adsorbent was added to 5 mL centrifugal tube containing 2 mL of ionic liquid solution. The following conditions of the adsorption experiments were applied: temperature in the range 5–90 °C, time in the range 0–30 min., IL concertation from 5 to 50 µg/mL. The tubes were shaken in a temperature-controlled shaker (Gallenkamp Orbital Incubator, Loughborough, UK) at a constant speed of 180 rpm. After that the mixture was centrifuged at 9000× *g*. An aliquot of the supernatant was further analysed by a HPLC procedure.

## 4. Conclusions

In this work, solid-liquid equilibria were determined and analyzed for systems composed of imidazolium and pyridinium ionic liquids and silica gel. It was found that imidazolium ionic liquids with a longer alkyl chain (BMIM) and a chaotropic anion (PF_6_^−^) with lower Gibbs free energy of hydration exhibited stronger adsorption ability in comparison to cations with shorter alkyl substituents: EMIM, EMPyr, EPyr and less chaotropic anions: Cl^−^, Br^−^, BF_4_^−^. Adsorption data fitting to Ho and Blanchard’ linear relationship: *t*/*q*(*t*) *vs. t* [37,38,39] enabled the selection of a pseudo-second-order kinetic model (PSO). Developed relationships could be used to extrapolate the kinetic data and estimate the values of *q*_e_ with a relative error of no more than 10%. Under the optimized conditions adsorption processes were not sensitive to the temperature in the range 5–40 °C, thus in practice they should be very effective media for the effective and economical recovery of ionic liquids from water at room temperature.

## References

[1] Lozinskaya E.I., Shaplov A.S., Kotseruba M.V., Komarova L.I., Lyssenko K.A., Antipin M.Y., Golovanov D.G., Vygodskii Y.S. (2006). “One-pot” synthesis of aromatic Poly(1,3,4-oxadiazole)s in novel solvents—Ionic liquids. J. Polym. Sci. A1.

[2] Shaplov A.S., Lozinskaya E.I., Odinets I.L., Lyssenko K.A., Kurtova S.A., Timofeeva G.I., Iojoiu C., Sanchez J.Y., Abadie M.J.M., Voytekunas V.Y. (2008). Novel phosphonated Poly(1,3,4-oxadiazole)s: Synthesis in ionic liquid and characterization. React. Funct. Polym..

[3] Matveeva E.V., Odinets I.L., Kozlov V.A., Shaplov A.S., Mastryukova T.A. (2006). Ionic-liquid-promoted Michaelis-Arbuzov rearrangement. Tetrahedron Lett..

[4] Vygodskii Y.S., Shaplov A.S., Lozinskaya E.I., Filippov O.A., Shubina E.S., Bandari R., Buchmeiser M.R. (2006). Ring-opening metathesis polymerization (ROMP) in ionic liquids: Scope and limitations. Macromolecules.

[5] Adams D.J., Dyson P.J., Taverner S.J. (2003). Chemistry in Alternative Reaction Media.

[6] Bharate J.B., Bharate S.B., Vishwakarma R.A. (2014). Metal-Free, Ionic Liquid-Mediated Synthesis of Functionalized Quinolines. ACS Comb. Sci..

[7] Cui X., Cai J., Zhang Y., Li R., Feng T. (2011). Kinetics of Transesterification of Methyl Acetate and n-Butanol Catalyzed by Ionic Liquid. Ind. Eng. Chem. Res..

[8] Gu Y., Shi F., Deng Y. (2004). Esterification of Aliphatic Acids with Olefin Promoted by Brønsted Acidic Ionic Liquids. J. Mol. Catal. A Chem..

[9] Xing H., Wang T., Zhou Z., Dai Y. (2005). Novel Brønsted-Acidic Ionic Liquids for Esterifications. Ind. Eng. Chem. Res..

[10] Wu Q., Chen H., Han M., Wang D., Wang J. (2007). Transesterification of Cottonseed Oil Catalyzed by Brønsted Acidic Ionic Liquids. Ind. Eng. Chem. Res..

[11] Qiao K., Yokoyama C. (2004). Nitration of Aromatic Compounds with Nitric Acid Catalyzed by Ionic Liquids. Chem. Lett..

[12] Xin H., Wu Q., Han M., Wang D., Jin Y. (2005). Alkylation of Benzene with 1-Dodecene in Ionic Liquids [Rmim]^+^Al_2_Cl_6_X^–^ (R = butyl, octyl and dodecyl; X = chlorine, bromine and iodine). Appl. Catal. A.

[13] Yoo K., Burckle E.C., Smirniotis P.G. (2002). Isobutane/2-Butene Alkylation Using Large-Pore Zeolites: Influence of Pore Structure on Activity and Selectivity. J. Catal..

[14] Flieger J., el Blicharska G., Czajkowska-Zelazko A. (2014). Ionic Liquids as Solvents in Separation Processes. Austin J. Anal. Pharm. Chem..

[15] Flieger J., Czajkowska-Żelazko A., Kokorin A. (2011). Ionic Liquids in Separation Techniques in Ionic Liquids: Applications and Perspectives.

[16] Han D., Row K.H. (2010). Recent Applications of Ionic Liquids in Separation Technology. Molecules.

[17] Jia Z., Yuan W., Sheng C., Zhao H., Hu H., Baker G.L. (2015). Optimizing the electrochemical performance of imidazolium-based polymeric ionic liquids by varying tethering groups. J. Polym. Sci. A1.

[18] Hu H., Yuan W., Jia Z., Baker G.L. (2015). Ionic liquid-based random copolymers: A new type of polymer electrolyte with low glass transition temperature. RSC Adv..

[19] Hu H., Yuan W., Lu L., Zhao H., Jia Z., Baker G.L. (2014). Low glass transition temperature polymer electrolyte prepared from ionic liquid grafted polyethylene oxide. J. Polym. Sci. A1.

[20] Shaplov A.S., Ponkratov D., Vlasov P., Lozinskaya E.I., Gumileva L.V., Surcin C., Morcrette M., Armand M., Aubert P.H., Vidal F. (2015). Ionic semi-interpenetrating networks as new approach for highly conductive and stretchable polymer materials. J. Mater. Chem. A.

[21] Shaplov A.S., Marcilla R., Mecerreyes D. (2015). Recent Advances in Innovative Polymer Electrolytes based on Poly(ionic liquid)s. Electrochim. Acta.

[22] Shaplov A.S., Ponkratov D.O., Aubert P., Lozinskaya E.I., Plesse C., Vidal F., Vygodskii Y.S. (2014). A first truly all-solid state organic electrochromic device based on polymeric ionic liquids. Chem. Commun..

[23] Shaplov A.S., Ponkratov D.O., Vlasov P.S., Lozinskaya E.I., Malyshkina I.A., Vidal F., Aubert P.H., Armand M., Vygodskii Y.S. (2014). Solid-state electrolytes based on ionic network polymers. Polym. Sci. Ser. B.

[24] Shaplov A.S., Ponkratov D.O., Aubert P., Lozinskaya E.I., Plesse C., Maziz A., Vlasov P.S., Vidal F., Vygodskii Y.S. (2014). Truly solid state electrochromic devices constructed from polymeric ionic liquids as solid electrolytes and electrodes formulated by vapor phase polymerization of 3,4-ethylenedioxythiophene. Polymer.

[25] Vygodskii Ya.S., Mel’nik O.A., Shaplov A.S., Lozinskaya E.I., Malyshkina I.A., Gavrilova N.D. (2007). Synthesis and ionic conductivity of polymer ionic liquids. Polym. Sci. Ser. A.

[26] Dadfarnia S., Shabani A.M., Bidabadi M.S., Jfari A.A. (2010). A novel ionic liquid/micro-volume back extraction procedure combined with flame atomic absorption spectrometry for determination of trace nickel in sample of nutritional interest. J. Hazard. Mater..

[27] Flieger J., Siwek A., Pizoń M., Czajkowska-Żelazko A. (2013). Ionic liquids as surfactants in micellar liquid chromatography. J. Sep. Sci..

[28] Dreyer S., Salim P., Kragl U. (2009). Driving forces of protein partitioning in an ionic liquid-based aqueous two-phase system. Biochem. Eng. J..

[29] Flieger J., Czajkowska-Żelazko A. (2015). Aqueous two phase system based on ionic liquid for isolation of quinine from human plasma sample. Food Chem..

[30] Liu J.F., Jiang G.B., Chi Y.G., Cai Y.Q., Zhou Q.X., Hu J.T. (2003). Use of ionic liquids for liquid-phase microextraction of polycyclic aromatic hydrocarbons. Anal. Chem..

[31] Freire M.G., Neves C.M.S.S., Marrucho I.M., Lopes J.N.C., Rebelo L.P.N., Coutinho J.A.P. (2010). High-performance extraction of alkaloids using aqueous two- phase systems with ionic liquids. Green Chem..

[32] Berton P., Monasterio R.P., Wuilloud R.G. (2012). Selective extraction and determination of vitamin B12 in urine by ionic liquid-based aqueous two-phase system prior to high-performance liquid chromatography. Talanta.

[33] Wang Y., Han Y.A., Xie X.Q., Li C.X. (2010). Extraction of trace acetylspiramycin in real aqueous environments using aqueous two-phase system of ionic liquid 1-butyl-3-methylimidazolium tetrafluoroborate and phosphate. Cent. Eur. J. Chem..

[34] Li C.X., Han J., Wang Y., Yan Y.S., Xu X.H., Pan J.M. (2009). Extraction and mechanism investigation of trace roxithromycin in real water samples by use of ionic liquid-salt aqueous two-phase system. Anal. Chim. Acta.

[35] Lui Q., Xuesheng H., Wang Y., Yang P., Xia H., Yu J., Liu H. (2005). Extraction of penicillin G by aqueous two-phase system of [Bmim]BF_4_/NaH_2_PO_4_. Chin. Sci. Bull..

[36] Fontanals N., Pocurull E., Marcé R.M., Borrull F., Mun J., Sim H. (2012). Handbook of Ionic Liquids. Properties, Applications and Hazards.

[37] Vidal M.-L., Riekkola A., Canals A. (2012). Ionic liquid-modified materials for solid-phase extraction and separation. Anal. Chim. Acta.

[38] Liu J., Li N., Jiang G., Liu J., Jönsson J.Å., Wen M. (2005). Disposable ionic liquid coating for headspace solid-phase microextraction of benzene, toluene, ethylbenzene, and xylenes in paints followed by gas chromatography-flame ionization detection. J. Chromatogr. A.

[39] Zhao F., Meng Y., Anderson J.L. (2008). Polymeric ionic liquids as selective coatings for the extraction of esters using solid-phase microextraction. J. Chromatogr. A.

[40] Liang P., Peng L. (2010). Ionic liquid-modified silica as sorbent for preconcentration of cadmium prior to its determination by flame atomic absorption spectrometry in water samples. Talanta.

[41] Stepnowski P., Muller A., Behrend P., Ranke J., Hoffmann J., Jastorff B. (2003). Reverse phase liquid chromatographic method for the determination of selected room temperature ionic liquids cations. J. Chromatogr. A.

[42] Kowalska S., Buszewski B., Stepnowski P. (2006). The influence of stationary phase properties on ionic liquid cations separation in RP-HPLC. J. Sep. Sci..

[43] Flieger J., Czajkowska-Żelazko A. (2012). Identification of ionic liquid components by RP-HPLC with diode array detector using chaotropic effect and perturbation technique. J. Sep. Sci..

[44] He H., Zhong M., Adzima B., Luebke D., Nulwala H., Matyjaszewski K. (2013). A Simple and Universal Gel Permeation Chromatography Technique for Precise Molecular Weight Characterization of Well-Defined Poly(ionic liquid)s. J. Am. Chem. Soc..

[45] (2005). Validation of Analytical Procedures: Text and Methodology.

[46] Marcus Y. (1991). Thermodynamics of Solvation of Ions. J. Chem. Soc. Faraday Trans..

[47] Ho Y.-S., Wase D., Forster C. (1996). Kinetic studies of competitive heavy metal adsorption by sphagnum moss peat. Environ. Technol..

[48] Ho Y.-S., McKay G. (1999). Pseudo-second order model for sorption processes. Process Biochem..

[49] Blanchard G., Maunaye M., Martin G. (1984). Removal of heavy metals from waters by means of natural zeolites. Water Res..

